# Simple and Complex Phonic Tics in Tourette Syndrome

**DOI:** 10.3390/brainsci15060620

**Published:** 2025-06-08

**Authors:** José Fidel Baizabal-Carvallo, Joseph Jankovic

**Affiliations:** 1 Parkinson’s Disease Center and Movement Disorders Clinic, Department of Neurology, Baylor College of Medicine, Houston, TX 77030, USA; 2 Department of Sciences and Engineering, University of Guanajuato, León 36000, Mexico

**Keywords:** Tourette syndrome, tics, vocal tics, phonic tics

## Abstract

Tourette syndrome (TS) is the most common cause of tics. Tics are classified as motor and phonic tics. The latter (previously also referred to as “vocal tics”) are manifested by simple sounds (simple phonic tics) or complex, often semantically meaningful utterances (complex phonic tics). Methods: We compared the clinical and demographic features of consecutive patients with TS who exhibited simple and complex phonic tics. Results: There were 149 patients, 117 (78.5%) of whom were males; the mean age at evaluation was 19.61 ± 12.97 years. In total, 35 (23.5%) of these manifested complex phonic tics, and 26 (17.4%) had verbalizations. No statistically significant differences were observed between TS patients with simple versus complex phonic tics with respect to sex, age at onset, age at presentation, or comorbid attention-deficit/hyperactivity disorder or obsessive–compulsive disorder. Patients with complex phonic tics more frequently had trunk tics (*p* = 0.002), complex motor tics (*p* < 0.001), copropraxia (*p* = 0.002), a wider variety of phonic tics (*p* < 0.001) and greater tic severity (*p* = 0.001). The multivariate regression analysis showed an independent association between trunk tics and complex phonic tics. Conclusions: Complex phonic tics seem to be part of a more widely distributed, severe, and complex presentation of TS, likely representing a continuum within the spectrum of motor and phonic tics.

## 1. Introduction

Tics are rapid, abrupt, repetitive movements (motor tics) or sounds (phonic tics) that may vary in frequency, intensity, and complexity within and among individuals [1]. Tics are classified as “simple motor tics” when muscle contractions lead to a visible jerk-like movement (“clonic tic”), more sustained movement (“dystonic tic”), sudden cessation of movement (“blocking tic”), or muscle contraction without visible movement (“isometric tic”) and are classified as “complex motor tics” when manifested by a sequence of several movements or repetitive, coordinated movements (“stereotypic tics”) or obscene gestures (“copropraxia”) [1,2]. Most patients with motor tics also have phonic tics such as single meaningless sounds or noises (e.g., sniffing, throat clearing, grunting), so called “simple phonic tics”, or semantically meaningful utterances (e.g., echolalia, palilalia, or shouting of obscenities, called coprolalia) [1,2]. The loudness of complex phonic tics may vary from mere whispers to loud shrieks, as noted in some patients referred to as “screamers”. Phonic tics are often described in the literature as “vocal tics”, but we prefer the term “phonic tics” because not all sounds involve vocal cords, such as sniffing, teeth clicking, sucking sounds using the tongue, or sounds produced by the lips (e.g., whistling, blowing, simulating flatulence, etc.) [1,2].

Most of these tics were recognized in a seminal monograph published in 1885 by Georges Albert Édouard Brutus Gilles de la Tourette, a French neurologist, who described nine patients with motor tics, six with phonic tics, five with coprolalia, and five with echolalia [3]. However, there are earlier accounts of the disorder that now bears Tourette’s name, Tourette syndrome (TS). The case of a priest who appeared to have motor and phonic tics and was thought to be possessed by the devil and engage in “witchcraft” was reported by Jakob Sprenger and Heinrich Kraemer in the book *Malleus Maleficarum*, published in 1498 [3]. In 1825, the French physician Jean-Gaspard Itard (1775–1838) described the case of the “Marquise de Dampierre”, who was actually “Countess Picot de Dampierre”, who exhibited sudden verbal outbursts consistent with coprolalia in salons frequented by the 19th-century Parisian aristocracy [4]. She was one of the nine patients described in the original publication by Tourette. Moreover, the Parisian physician Armand Trousseau described several cases of motor and phonic tics 12 years before Tourette’s seminal description [5,6]. These historical accounts highlight the importance of “phonic” tics in the repertoire of tics manifested by patients with TS.

Involuntary vocalizations, characteristic of tics in patients with TS, have also been described in patients with a variety of other neurological disorders, including Parkinsonian disorders, Huntington’s disease, and many other conditions. [7]. According to the DSM-5 [8], phonic tics must be present in order to make a TS diagnosis. This criterion, however, is quite arbitrary, as most phonic tics are actually motor tics in which muscle contractions of nasal, oral, pharyngeal, laryngeal, chest, and other articulatory structures result in sounds. This artificial diagnostic criterion was addressed in a recent “reappraisal” which mandated the presence of “motor and/or phonic tics” [9]. Unfortunately, the revised criteria, which are otherwise quite similar to the DSM-5 criteria, insisted that in order to make a TS diagnosis, the tics must “cause some degree or impairment at any point in time”. This does not take into account the fact that many patients with TS are not bothered by their tics, perhaps finding them embarrassing or even troublesome but not necessarily “impairing”. A list of phonic tics is provided in Table 1.

Despite the many clinical descriptions reported in the medical literature [1,2,3,7], it is unclear whether simple and complex phonic tics represent a fundamentally different phenomenology or are part of a continuum of motor and phonic tics. We aimed to assess these clinical features and evaluate patients with simple versus complex phonic tics.

## 2. Patients and Methods

We performed a retrospective study of the medical charts and video recordings of 149 consecutive patients meeting the diagnostic criteria of TS according to the Diagnostic and Statistical Manual of Mental Disorders 5th edition (DSM-5) [8], who presented for evaluation at the Movement Disorders Clinic of Baylor College of Medicine in Houston, Texas. We excluded patients with secondary tic disorders, provisional tic disorder, and chronic (persistent) motor or phonic tics according to the DSM-5. All patients were videotaped according to a standardized protocol reported elsewhere [10]. Briefly, the recording included an interview regarding the tics previously and currently affecting the patient, followed by a neurological examination focusing on capturing any abnormal movements or emitted sounds. During the course of the recording, the videographer stepped out of the video studio while the camera was running in order to capture tics that would otherwise be suppressed by the patient in the presence of the examiner. The video recordings typically lasted between 15 and 20 min.

We assessed demographic variables (sex, age at onset, and age at evaluation), tic type, and body distribution of tics. All enrolled patients had a history of at least one phonic tic (as required for the diagnosis of TS), but not all of them exhibited phonic tics during the video recordings. The complexity of phonic tics was classified according to Table 1.

Videos were initially analyzed by a group of 6 neurologists specializing in movement disorders, and clinical data was registered for each patient. Additionally, video recordings were analyzed for tic severity based on the Rush Video-Based Tic Rating Scale by the first author of this study, a neurologist specializing in movement disorders with more than 15 years of clinical experience in the assessment of movement disorders. The scores on the Rush Video-Based Tic Rating Scale range from 0 to 6 [11], categorizing the presence of tics as follows: 0: none; 1: very mild (tics uncommonly seen on video); 2: mild (tics are common and noticeable but not disruptive); 3: medium (tics are common and mildly disruptive); 4: marked (tics are common and moderately disruptive); 5: severe (tics are very common and moderately disruptive); and 6: very severe (tics are very common and extremely disruptive). We divided patients into two groups, one with simple phonic tics only and one comprising patients with at least one complex phonic tic; this group may or may not have had comorbid simple phonic tics. For this study, we assessed whether patients had “verbalizations” as part of their complex phonic tics. We defined “verbalization” as patients uttering a full meaningful word, phrase, or sentence.

Neuropsychiatric comorbidities including attention-deficit/hyperactivity disorder (ADHD) and obsessive–compulsive disorder (OCD) were also documented in each patient according to the DSM-5 criteria [8]. This study was performed according to the Declaration of Helsinki. Patients or a family member provided signed written informed consent, approved by the Baylor College of Medicine Institutional Review Board for Human Research for videotaping and publishing in a scientific journal according to the Health Insurance Portability and Accountability Act (HIPAA) of 1996. This study was conducted in accordance with the Declaration of Helsinki. The Internal Review Board of Santé Medical Tower approved the protocol on 21 May 2025 with the reference number 25053C.

### Statistics

Statistical evaluations were performed using SPSS version 22. We summarized data in percentages, means, and standard deviations. The chi-square test with Yates’s continuity correction or Fisher’s exact tests were performed to compare ordinal data between groups. The *t*-test for independent samples was used to compare means between groups. We performed a multivariate logistic regression analysis using a backward stepwise Wald method. Significant variables in the bivariate analysis were selected as independent variable with the presence of “complex phonic” tics as the dependent variable. Odds ratios (ORs) with a 95% confidence interval (C.I.) were calculated using the (exp)^B^ coefficients. Goodness of fit was estimated with the Hosmer–Lemeshow test, and the coefficient of determination (R^2^) of the regression was calculated with the Nagelkerke test. A *p*-value < 0.05 was considered significant.

## 3. Results

There were 149 patients with TS, 117 males and 32 females with a mean (±S.D.) age of 19.61 ± 12.97 years at evaluation and mean (±S.D.) age of 8.58 ± 8.57 years at onset. Among these patients, 35 (23.5%) were identified as having complex phonic tics, and 26 (17.4%) were identified as having verbalizations. Some patients with complex phonic tics also exhibited echolalia (*n* = 7, 20%), palilalia (*n* = 2, 5.7%), and coprolalia (*n* = 17, 48.6%). No differences were identified in sex distribution, age at onset, and at evaluation between patients with simple and complex phonic tics (Table 2).

The distribution of tics did not differ between patients with simple and complex phonic tics, except for arm (*p* = 0.026) and trunk tics that were significantly more frequently observed in patients with complex phonic tics (*p* ≤ 0.001). Moreover, complex motor tics and copropraxia were significantly more frequent in those with complex phonic tics (*p* ≤ 0.001). The number of phonic tics and the severity of tics was greater in patients with complex phonic tics. However, comorbid ADHD and OCD were not more common in patients exhibiting complex phonic tics. The differences in demographic and clinical features were essentially unchanged when contrasting patients with simple phonic tics with all patients with complex phonic tics or the subgroup with verbalizations (Table 2). In the multivariate logistic regression analysis, a greater number of phonic tics was associated with complex phonic tics and verbalizations, and the presence of trunk tics was independently associated with verbalizations (Table 3).

## 4. Discussion

In this study, we compared the demographic and clinical features of TS patients with simple phonic tics versus those also showing complex phonic tics; the latter were also subdivided into those with and without verbalizations. We found that patients with complex phonic tics more frequently had trunk tics, complex motor tics, copropraxia, greater tic severity, and a more extensive repertoire of phonic tics. These differences essentially remained unchanged when only considering patients with verbalizations, while no differences were observed in sex distribution, age at onset or evaluation, or comorbid ADHD and OCD.

The significant association of complex phonic tics with the presence of trunk tics (*p* = 0.013) is intriguing (Table 3). We hypothesize that the muscle activation used to produce complex phonic tics overlaps with respiratory and thoracic muscles involved in this tic distribution. Trunk tics may cause also breathing interference and can be confused with pulmonary disorders [12].

Patients with complex phonic tics more frequently have complex motor tics, including copropraxia, suggesting that those patients tend to have greater tic complexity overall, not only for audible sounds but also for motor behaviors. Additionally, tic severity was greater in those with complex phonic tics with a higher diversity of phonic tics. This latter finding was independently associated with complex phonic tics after controlling for other variables showing statistical significance in the bivariate analysis. This evidence suggests that patients with complex phonic tics have greater severity, variability, and complexity of the underlying tic disorder than those with simple phonic tics. However, the lack of differences in variables such as sex, age, comorbid ADHD, or OCD between patients with simple and complex phonic tics suggests that these tics more likely represent a continuum in phenomenology severity and complexity between these groups. This is in accordance with previous studies where no major differences were noted in patients with TS + ADHD vs. TS only [13]. In the case of OCD, an initial multivariate study reported that patients with TS + OCD were more commonly male, had an early age at onset, and had greater aggressive obsessions, cleaning compulsions, and trichotillomania [14]; however, these findings were not confirmed in a later study [15]. Further studies should help to clarify whether different tic phenomenology can be observed in patients with comorbid OCD and ADHD.

It is unclear why the phonatory apparatus is particularly involved in patients with TS. It has been proposed that phonic tics and motor tics involving the cranial and axial muscles may be related to the social behavior network (SBN), a group of structures which interconnects the basal ganglia, the amygdala, periaqueductal gray matter, and other relevant limbic structures [16]. The SBN helps to mediate sex-specific and socially pertinent phonations and facial expressions in certain animal species and humans [16]. This may explain why patients with complex phonic tics exhibit a larger number of phonic tics and greater complexity of motor tics, including more frequent coprophenomena (i.e., copropraxia and coprolalia) as observed in our study.

One experimental study in monkeys showed bilateral limbic cortical–subcortical activation with disinhibition of the nucleus accumbens (NAc) and with abnormal, but inconsistent, spikes in local field potentials (LFPs) detected in this structure and the anterior cingulate cortex (ACC), along with phase–phase alpha oscillation coupling between the NAc, the ACC, and the primary motor cortex, preceding sound emissions [17]. Moreover, premovement potentials were recorded in the cingulate and prefrontal cortices in monkeys who exhibited uttering with intense motivational rewards [18]. These areas are readily involved in emotional and motivational processing [19].

These findings highlight the role of limbic structures, coupled with motor networks in the generation and persistence of phonic tics. This seems to contrast with clonic motor tics, where participation of limbic structures is modest, but there is more robust activation of the ipsilateral sensorimotor system observed in PET scans [20]. The experimental disinhibition model in monkeys, using injections of bicuculline in the sensorimotor putamen, showed the appearance of periodic tics in the orofacial muscles [20]. This occurs with a 64% increased activity of neurons in the cerebellar cortex and a variety of responses in the neurons of the dentate nuclei [20]. This finding highlights the emerging role of the cerebellum in the pathogenesis of tics, where, along with the primary motor cortex, it seems to have a function as a gate for tic release [21]. The question of how the cerebellum may influence the occurrence of phonic tics is unclear; however, right cerebellar hemispherectomy in monkeys eliminated premovement potentials in the motor cortex and in the posterior bank of an area homolog of human Broca’s area in the left hemisphere in monkeys, changing motor and vocal expressions [18].

Coprolalia, one of the most over-emphasized features of TS, is a classic example of a complex phonic tic, highlighted in Tourette’s seminal 1885 paper [3]. Coprolalia may sometimes be difficult to assess, as it may present as slurred or partially pronounced words or disguised in neologisms or euphemisms [22]. It may also be denied by patients or family members due to its stigmatizing character. Moreover, some patients may also think of the obscene word without uttering it (mental coprolalia). In a series of 558 consecutive new cases of TS from 15 sites and seven countries, coprolalia occurred at some point in 19.3% of males and 14.6% of females at a mean age at onset of 11 years; in 11%, coprolalia was the initial symptom of TS [23]. Coprolalia is usually in a form of uttering (swearing) obscenities (foul, repulsive language often with sexual or scatological meaning), rather than profanities (cursing with religious meaning), although some have racist, sexist, or vulgar (coarse or crude) meaning. Coprolalia as part of coprophenomena has been related to higher tic severity scores and poor global and family functioning in individuals suffering from it [24]. A coprophenomenon, often coupled with impulsive behavior, rage outbursts, intrusive behavior (e.g., forced touching), alcohol, and substance abuse may trigger or eventually lead to TS patients becoming involved with law enforcement and the legal system [25,26,27].

Coprophenomena, which rarely occurs in other conditions, such as neuroacanthocytosis [28], is not well understood but may be an example of prominent disinhibition in neural pathways linking the cortico-basal ganglia-thalamic circuits and the limbic structures. However, the underlying pathophysiology is difficult to assess, as there are no animal models of coprolalia and pathophysiology studies should rely on human studies. Coprolalia has been reported to improve with the D2-receptor partial agonist aripiprazole and botulinum toxin (BoNT) injections in the vocal cords [29,30].

Tics observed in the context of TS should be differentiated from functional tics. There is significant overlap between both types of tics, as both can show some degree of distractibility and suggestibility, with bouts of tics. Moreover, patients with functional tics present a more restricted body distribution, with a less complex and less severe syndrome compared to those with TS [31]. However, patients with functional tics usually deny having a premonitory feeling, have an older age at onset, and are unable to transitorily suppress their tics. However, these features have not been readily evaluated for phonic tics.

Our study has limitations, as it was conducted in a tertiary care center for TS. It is possible that enrolling patients in a referral center for movement disorders might have introduced a selection bias of patients towards those with greater TS severity. However, the recruited patients had diverse degrees of tic severity, making the cohort representative of patients with TS. Another limitation is the lack of longitudinal data, making it not possible to determine the time at onset of complex phonic tics during the evolution of the condition. Because of the retrospective nature of this study, we were not able to determine the frequency or nature of premonitory urges commonly preceding motor as well as phonic tics [32]. We did not determine the presence of other comorbid neuropsychiatric disorders including ODD, depression, and anxiety in order to determine if they were fundamentally different between patients with simple and complex phonic tics. Another limitation is that we did not assess the effect of therapy in our cohort of patients. It has been observed that pharmacological therapies aiming to decrease motor tics have beneficial effects on phonic tics; moreover, the use of (BoNT) in the laryngeal muscles has shown to be useful in decreasing all types of phonic tics, including coprolalia or complex vocalizations [33].

## 5. Conclusions

In summary, patients with complex phonic tics were more frequently associated with trunk tics and a greater number of phonic tics. The latter suggests that these patients had more extensively distributed tics with greater severity, variability, and complexity than patients exhibiting only simple phonic tics. However, simple and complex phonic tics, including verbalizations, seem to be part of a continuum of tic severity and complexity.

## Figures and Tables

**Table 1:** Examples of simple and complex phonic tics.

Simple	Complex
Belching Clicking Coughing Gasping Grunting Gurgling Guttural sounds Hiccupping Hissing Honking Moaning Noisy breathing Puffing Screaming Sniffing Snorting Squeaking Squealing Sucking Throat clearing ‘tsk’, ‘pft’, etc. Yelping	Saying: Syllables Partial words Partial echolalia Partial palilalia Partial coprolalia Full words * Full sentences * Echolalia * Palilalia * Coprolalia *

* These are considered “verbalizations” in the present study.

**Table 2:** Contrasting features between patients with simple vs. complex phonic tics.

	Simple Phonic tics, *n* = 114	Complex Phonic Tics, *n* = 35	Verbalizations ^†^, *n* = 26	*p*-Value (Simple vs. Complex Phonic Tics)	*p*-Value (Simple vs. Verbalizations)
Sex (Male)	88 (77.2%)	29 (82.9%)	21 (80.8%)	0.632	0.893
Age at onset (years) (SD)	8.34 ± 8.45	9.25 ± 9.05	10.20 ± 10.09	0.620	0.372
Age at examination (years) (SD)	20.18 ± 13.65	17.74 ± 10.38	17.92 ± 11.14	0.264	0.377
Head/facial tics	99 (86.4%)	32 (91.4%)	23 (88.5%)	0.666	1.000
Head jerks	69 (60.5%)	25 (71.4%)	19 (73.1%)	0.333	0.332
Arm tics	41 (36%)	20 (57.1%)	15 (57.7%)	0.026	0.069
Shoulder tics	50 (43.9%)	18 (51.4%)	15 (57.7%)	0.351	0.214
Trunk tics *	32 (28.1%)	20 (57.1%)	18 (69.2%)	0.002	<0.001
Leg tics	30 (26.3%)	13 (37.1%)	12 (46.2%)	0.216	0.079
Complex motor tics *	49 (42%)	29 (82.9%)	21 (80.7%)	<0.001	0.001
Copropraxia *	3 (2.6%)	7 (20%)	5 (19.2%)	0.002	0.006
Dystonic tics	26 (22.8%)	9 (26.7%)	9 (34.6%)	0.899	0.210
ADHD	46 (40.4%)	20 (57.1%)	13 (50%)	0.120	0.497
OCD	61 (53.5%)	23 (65.7%)	17 (65.4%)	0.244	0.310
Number of phonic tics * (mean, SD)	2.19 ± 1.47	4.63 ± 0.97	4.65 ± 0.85	<0.001	<0.001
Tic severity score * (mean, SD)	3.25 ± 1.05	3.94 ± 1.11	3.92 ± 1.13	0.001	0.004

ADHD: attention-deficit/hyperactivity disorder; OCD: obsessive–compulsive disorder. * Statistically significant. ^†^ Verbalization was considered when patients uttered a full meaningful word, phrase, or sentence.

**Table 3:** Multivariate regression analysis showing independent variables associated with complex phonic tics and verbalizations.

	B Coefficient	Wald	OR (ExpB)	95% C.I. ExpB	*p*-Value
Complex phonic tics (M1)					
Copropraxia	0.834	0.808	2.303	0.373–14.198	0.369
Tic severity	0.275	1.251	1.317	0.813–2.132	0.263
Arm tics	−0.701	1.260	0.496	0.146–1.687	0.262
Complex tics	0.798	1.725	2.220	0.675–7.299	0.189
Number of phonic tics *	1.134	29.92	3.107	2.070–4.664	<0.001
Trunk tics	0.911	3.13	2.488	0.906–6.834	0.077
Verbalizations (M2)					
Tic severity	0.015	0.003	1.015	0.603–1.708	0.955
Complex tics	0.296	0.181	1.345	0.344–5.262	0.670
Copropraxia	0.970	0.848	2.638	0.335–20.807	0.357
Number of phonic tics *	1.166	22.22	3.209	1.976–5.210	<0.001
Trunk tics *	1.449	6.16	4.259	1.356–13.375	0.013

Model 1 (M1): Hosmer–Lemeshow: *p* = 0.942, Nagelkerke: R^2^ = 0.537. Model 2 (M2): Hosmer–Lemeshow: *p* = 0.842, Nagelkerke: R^2^ = 0.552. * Statistically significant.

## Data Availability

The original contributions presented in this study are included in the article. Further inquiries can be directed to the corresponding author.
